# Knowledge, attitudes, and practices toward artificial intelligence among health sciences students: A cross-sectional study in Palestine

**DOI:** 10.1371/journal.pone.0352785

**Published:** 2026-06-26

**Authors:** Nesreen Alqaissi, Mohammad Qtait, Khalaf Awwad, Zeenat Mesk, Fuad Farajalla, Yasmeen Ziad, Farah Abu-Salameh, Amani Hmedat, Rawan Halayka, Maysam Ajlouni, Yasmeen Shareef

**Affiliations:** Department of Nursing, College of Nursing, Palestine Polytechnic University, Hebron, Palestine; Freelance Consultant, Myanmar, MYANMAR

## Abstract

**Background:**

Artificial intelligence (AI) is increasingly integrated into healthcare education and clinical practice. Understanding health sciences students’ knowledge, attitudes, and practices (KAP) toward AI is important for informing curriculum development, particularly in resource-limited educational settings.

**Objective:**

To assess knowledge, attitudes, and practices toward artificial intelligence among health sciences students at a Palestinian university.

**Methods:**

A cross-sectional study was conducted during the 2024–2025 academic year among 666 undergraduate students from nursing, medicine and health sciences, and dentistry programs. Data were collected using a structured questionnaire assessing AI knowledge (7 items), attitudes (10 items), and practices (7 items). Descriptive statistics were calculated. Independent-samples t-tests and one-way analysis of variance (ANOVA) were used to examine group differences. Effect sizes were reported using Cohen’s d and eta squared (η²). Statistical significance was set at p < 0.05.

**Results:**

The overall AI knowledge accuracy rate was 42.9% (mean 3.00 ± 1.61), indicating limited foundational understanding, particularly regarding machine learning and deep learning concepts. Attitudes toward AI were generally positive (mean 3.60 ± 0.44), with high endorsement of ethical awareness. AI practice levels were moderate (mean 3.32 ± 0.70), with frequent use for assignments and research activities. Formal AI training was associated with higher knowledge scores (t(664)=7.45, p < 0.001, d = 0.69) and slightly more positive attitudes (t(664)=2.59, p = 0.010, d = 0.23), but not with practice frequency (p = 0.807). Differences across colleges and academic years demonstrated small effect sizes (η² ≤ 0.03).

**Conclusion:**

Health sciences students demonstrated positive attitudes and regular academic use of AI tools despite limited foundational knowledge. These findings suggest the potential benefit of structured AI literacy integration within health sciences curricula.

## Introduction

Artificial intelligence (AI) has rapidly emerged as a transformative force across healthcare systems worldwide, reshaping clinical decision-making, education, diagnostics, and health service delivery [1,2]. Advances in machine learning, natural language processing, and generative AI have enabled sophisticated applications ranging from clinical decision support systems to personalized learning platforms and intelligent tutoring tools [3,4]. Within health professions education, AI has demonstrated potential to enhance students’ critical thinking, academic performance, and access to evidence-based information, while simultaneously raising ethical, professional, and pedagogical concerns [5–7].

Health sciences students represent a critical population in the successful integration of AI into future healthcare practice. As future nurses, physicians, and allied health professionals, students’ knowledge, attitudes, and practices toward AI influence not only their educational outcomes but also their readiness to adopt AI-enabled technologies in clinical environments [8]. Evidence suggests that insufficient AI literacy among healthcare students may hinder effective and ethical AI implementation, potentially exacerbating digital inequities and compromising patient safety [9,10]. Consequently, international organizations, including the World Health Organization, emphasize the urgent need to integrate AI competencies into health professions curricula to prepare a digitally competent workforce [11].

Despite growing interest, existing literature indicates considerable variability in students’ understanding and acceptance of AI. Studies conducted in Europe, Asia, and the Middle East report generally positive attitudes toward AI in healthcare education, alongside limited formal training and fragmented knowledge [12–14]. Nursing students, in particular, often demonstrate enthusiasm toward AI-supported learning tools such as ChatGPT, simulation platforms, and decision-support applications, yet simultaneously express concerns related to data privacy, ethical accountability, over-reliance on technology, and the potential erosion of the humanistic aspects of care [15,16]. These mixed perceptions underscore the importance of systematically assessing students’ knowledge, attitudes, and practices to inform curriculum development and policy decisions.

The rapid diffusion of artificial intelligence has also generated societal ambivalence. Historical analyses suggest that technological transitions are often accompanied by fear, ethical concern, and uncertainty regarding professional identity and role displacement [17–19]. Such reactions may function not merely as resistance but as constructive mechanisms that stimulate ethical reflection, governance, and responsible innovation. Within health professions education, students’ mixed perceptions of AI simultaneous enthusiasm and concern should therefore be interpreted within broader sociocultural responses to technological change.

The Knowledge, Attitudes, and Practices (KAP) framework offers a robust theoretical and methodological approach for examining how individuals understand, perceive, and utilize emerging technologies such as AI [19]. Within health education research, the KAP model facilitates the identification of educational gaps, behavioral patterns, and contextual factors influencing technology adoption [20]. Applying this framework to AI in health sciences education allows educators and policymakers to move beyond descriptive attitudes toward actionable insights that can guide targeted interventions, training programs, and ethical guidelines.

In the Palestinian context, the integration of AI into higher education and healthcare remains at an early stage, constrained by infrastructural limitations, resource scarcity, and ongoing sociopolitical challenges [21]. Palestinian universities are increasingly adopting digital learning platforms and blended teaching approaches; however, structured AI education within health sciences curricula remains limited. At the same time, Palestinian healthcare systems face substantial workforce shortages, high patient-to-provider ratios, and growing demands for efficiency and quality improvement—conditions under which AI-supported solutions may offer significant benefits if appropriately implemented [22,23]. Understanding students’ readiness to engage with AI is therefore essential for aligning educational strategies with national healthcare needs.

Notably, there is a paucity of empirical research examining AI-related knowledge, attitudes, and practices among health sciences students in Palestine. Most regional studies originate from Gulf countries or broader Arab multi-country surveys, which may not fully capture the unique educational, cultural, and systemic characteristics of Palestinian institutions [14,24]. Addressing this gap is crucial to ensuring contextually relevant AI education that supports ethical practice, professional competence, and patient-centered care.

Accordingly, this study aims to assess the knowledge, attitudes, and practices toward artificial intelligence among health sciences students at a Palestinian university. By identifying key demographic and educational factors associated with AI perceptions and use, the study seeks to generate evidence that can inform curriculum reform, faculty development, and institutional policies. Ultimately, strengthening AI literacy among future health professionals is essential for harnessing the benefits of AI while safeguarding professional values, ethical standards, and the quality of healthcare delivery.

## Materials and methods

### Study design and reporting standards

An institutional-based cross-sectional study design was employed to assess health sciences students’ knowledge, attitudes, and practices (KAP) toward artificial intelligence (AI). The study was conducted and reported in accordance with the Strengthening the Reporting of Observational Studies in Epidemiology (STROBE) Statement for cross-sectional studies. A completed STROBE checklist is provided as supplementary material.

### Study setting

The study was conducted at Palestine Polytechnic University (PPU), Hebron, Palestine, during the second semester of the 2024–2025 academic year (March 20 to May 30, 2025). PPU is a public higher education institution offering undergraduate programs in Nursing, Medicine and Health Sciences, and Dentistry.

### Study population and eligibility criteria

The source population consisted of all undergraduate students enrolled in health-related programs at PPU during the study period. Inclusion criteria were:

1. Enrollment in the College of Nursing, College of Medicine and Health Sciences, or College of Dentistry;
2. Completion of at least one academic semester;
3. Age ≥ 18 years;
4. Provision of informed consent.

Students on academic leave were excluded.

#### Sampling strategy and participant recruitment.

A non-probability convenience sampling approach was used due to feasibility considerations and the exploratory nature of the study. All eligible students present during scheduled academic sessions were invited to participate.

A total of 742 students were approached. Of these, 681 agreed to participate (response rate = 91.8%). After excluding 15 questionnaires with substantial missing data (>20% incomplete responses), the final analytic sample consisted of 666 students.

### Sample size considerations

Although no prior Palestinian data were available to inform formal sample size estimation, the final sample (N = 666) exceeded the minimum number required to detect small-to-moderate effect sizes at 80% statistical power with α = 0.05 for between-group comparisons.

### Data collection instrument

Data were collected using a structured, self-administered questionnaire adapted from the artificial intelligence knowledge, attitudes, and practices instrument developed by Sallam, et,. (2023) [24] among health students. Permission to use and adapt the instrument was obtained from the original authors. Minor contextual adaptations were made to ensure relevance to the Palestinian health sciences education context, without modifying the conceptual structure or domain content of the original instrument.

The questionnaire comprised four domains:

#### Demographic and academic characteristics.

Twelve items assessed age, gender, college affiliation, academic year, and prior exposure to AI-related education or training.

#### Knowledge of artificial intelligence.

The knowledge domain included seven dichotomous items assessing foundational AI literacy, including:

- Understanding of AI basics
- Knowledge of machine learning and deep learning
- Awareness of AI applications in healthcare
- Understanding of AI data requirements
- Awareness of implementation barriers
- Exposure to AI training

Correct responses were scored as 1 and incorrect responses as 0. Total knowledge scores ranged from 0 to 7. Higher scores indicated greater AI knowledge. Overall knowledge accuracy was calculated as the percentage of correct responses across items.

Internal consistency was assessed using Kuder–Richardson Formula 20 (KR-20).

#### Attitudes toward artificial intelligence.

Attitudes were assessed using 10 Likert-scale items rated from 1 (strongly disagree) to 5 (strongly agree).

Items assessed both positive perceptions (e.g., AI education importance, future professional relevance) and concern-based perceptions (e.g., AI replacing professionals, diagnostic errors). Negatively framed items (Items 5, 8, 9, and 10) were reverse coded prior to computing the composite score to ensure that higher total scores consistently reflected more positive attitudes toward AI integration.

Total attitude scores ranged from 10 to 50.

Internal consistency was evaluated using Cronbach’s alpha.

#### Practices related to artificial intelligence.

The practice domain included seven Likert-scale items rated from 1 (never) to 5 (all the time), assessing frequency of AI use for:

- Examination preparation
- Assignments
- Research
- Brainstorming
- Career guidance
- Language support
- Skill development

Total scores ranged from 7 to 35, with higher scores indicating greater AI engagement.

### Instrument validity and reliability

#### Content validity.

Content validity was established through evaluation by five experts in nursing education, medical education, and digital health. Experts assessed clarity, cultural relevance, and conceptual alignment. Minor wording revisions were implemented following consensus feedback.

#### Pilot testing.

A pilot study was conducted among 35 health sciences students who were not included in the final sample. The pilot assessed clarity, feasibility, and reliability.

#### Reliability.

Domain-specific reliability coefficients were:

- Knowledge: KR-20 = 0.78
- Attitudes: Cronbach’s α = 0.84
- Practices: Cronbach’s α = 0.81

These values indicate acceptable internal consistency.

Item-total correlation analysis demonstrated corrected correlations above 0.30 for all scale items, supporting internal construct consistency.

### Data collection procedure

Data were collected using both paper-based and electronic questionnaires during scheduled academic sessions. Participation was voluntary and anonymous. No incentives were provided.

### Ethical considerations

Ethical approval was obtained from the Ethical Committee of the College of Nursing, Palestine Polytechnic University (Approval No. EA/2025/55). The study adhered to the Declaration of Helsinki principles.

Participants were informed about study objectives, confidentiality, and voluntary participation. Written informed consent was obtained. No identifying information was collected.

### Statistical analysis

Data were analyzed using IBM SPSS Statistics version 27.

#### Data preparation.

Data were screened for completeness and accuracy. Questionnaires with more than 20% missing responses were excluded. Remaining missing values (<2%) were handled using pairwise deletion.

Negatively worded attitude items were reverse coded prior to composite score calculation.

#### Descriptive statistics.

Categorical variables were summarized using frequencies and percentages. Continuous variables were summarized using means and standard deviations.

#### Assumption testing.

Normality of continuous variables was assessed using Shapiro–Wilk tests and visual inspection of Q–Q plots. Homogeneity of variance was evaluated using Levene’s test.

Given the large sample size (N = 666), parametric tests were retained based on their robustness to mild deviations from normality.

#### Inferential statistics.

- Independent-samples t-tests were used for two-group comparisons (e.g., gender, formal AI training).
- One-way ANOVA was used for multi-group comparisons (e.g., academic year, college affiliation).
- Bonferroni-adjusted post hoc tests were conducted where appropriate.
- Effect sizes were reported using Cohen’s d (t-tests) and eta squared (η²) for ANOVA.

All tests were two-tailed. Statistical significance was set at p < 0.05.

## Result

### Participant characteristics

A total of 666 health sciences students were included in the final analysis. The majority were female (n = 492, 73.9%). Participants were distributed across academic years, with the largest proportions in the second year (26.1%) and first year (24.3%). College representation included Nursing (36.3%), Medicine and Health Sciences (36.8%), and Dentistry (26.9%). Approximately 23.7% (n = 158) reported prior formal training in artificial intelligence (AI).

Descriptive statistics and group comparisons for knowledge, attitudes, and practices are presented in Table 1.

**Table 1:** Sociodemographic characteristics and differences in knowledge, attitudes, and practices toward artificial intelligence (N = 666).

Variable	Category	n (%)	Knowledge Mean ± SD	p value	Attitude Mean ± SD	p value	Practice Mean ± SD	p value
**Gender**	Female	492 (73.9)	2.94 ± 1.61	.182	3.58 ± 0.45	.937	3.28 ± 0.71	.106
	Male	174 (26.1)	3.15 ± 1.60		3.57 ± 0.43		3.36 ± 0.69	
**Academic Year**	First	162 (24.3)	2.68 ± 1.55	.006	3.56 ± 0.46	.215	3.32 ± 0.68	.476
	Second	174 (26.1)	3.24 ± 1.63		3.51 ± 0.41		3.31 ± 0.72	
	Third	153 (23.0)	3.22 ± 1.58		3.60 ± 0.43		3.40 ± 0.69	
	Fourth	131 (19.7)	2.89 ± 1.61		3.64 ± 0.44		3.26 ± 0.71	
	Fifth–Sixth	46 (6.9)	2.83 ± 1.67		3.68 ± 0.48		3.31 ± 0.75	
**College**	Nursing	242 (36.3)	2.81 ± 1.61	.029	3.62 ± 0.46	.034	3.18 ± 0.70	<.001
	Medicine & Health Sciences	245 (36.8)	3.19 ± 1.64		3.59 ± 0.41		3.38 ± 0.68	
	Dentistry	179 (26.9)	3.01 ± 1.54		3.51 ± 0.44		3.44 ± 0.72	
**Formal AI Training**	Yes	158 (23.7)	3.80 ± 1.52	<.001	3.65 ± 0.42	.010	3.30 ± 0.69	.807
	No	508 (76.3)	2.73 ± 1.58		3.55 ± 0.45		3.23 ± 0.71	

### Knowledge of artificial intelligence

The overall mean knowledge score was 3.00 ± 1.61 (range 0–7), corresponding to an accuracy rate of 42.9%. As shown in Table 2, high levels of correct responses were observed for awareness of AI applications in participants’ own fields (74.5%) and understanding AI barriers in healthcare (66.5%). In contrast, low correct response rates were reported for AI basics (32.0%) and machine/deep learning concepts (26.0%).

**Table 2:** Knowledge of artificial intelligence among health sciences students.

Item	Knowledge Statement	Correct n (%)	Incorrect n (%)
1	Knowledge of AI basics	213 (32.0)	453 (68.0)
2	Understanding of machine/deep learning	173 (26.0)	493 (74.0)
3	Awareness of AI applications in own field	496 (74.5)	170 (25.5)
4	Attendance of AI courses/workshops	127 (19.1)	539 (80.9)
5	AI taught during undergraduate study	154 (23.1)	512 (76.9)
6	Understanding data requirements of AI	393 (59.0)	273 (41.0)
7	Understanding AI barriers in healthcare	443 (66.5)	223 (33.5)
**Total Knowledge Accuracy**	—	**42.9%**	57.1%

No statistically significant gender differences were observed in knowledge scores, t(664) = 1.34, p = .182, Cohen’s d = 0.13.Knowledge differed significantly across academic years, F(4, 661) = 3.65, p = .006, η² = 0.02, indicating a small effect size.

College affiliation was significantly associated with knowledge scores, F(2, 663) = 3.58, p = .029, η² = 0.01.

Students with formal AI training had significantly higher knowledge scores than those without training, t(664) = 7.45, p < .001, Cohen’s d = 0.69 (moderate-to-large effect).

### Attitudes toward artificial intelligence

After reverse coding negatively framed items, the overall mean attitude score was 3.60 ± 0.44, reflecting generally positive perceptions toward AI integration Table 3.

**Table 3:** Attitudes toward artificial intelligence integration.

Item	Statement	Mean	SD
1	Healthcare students should learn AI basics	4.09	0.75
2	AI will be essential in my profession	3.95	0.83
3	Ethical implications must be understood	4.26	0.76
4	AI will revolutionize education	4.04	0.79
5	AI will replace human teachers	2.60	1.07
6	Future AI developments excite me	3.56	0.87
7	AI should be part of health training	3.75	0.89
8	Some specialties are replaceable by AI	3.26	1.07
9	Clinical AI more accurate than physicians	2.62	1.09
10	AI increases diagnostic errors	3.67	1.04
**Overall Attitude Score**	—	**3.60**	0.44

No significant gender differences were identified, t(664) = 0.08, p = .937. Attitude scores did not significantly differ by academic year, F(4, 661) = 1.46, p = .215. College affiliation was significantly associated with attitudes, F(2, 663) = 3.42, p = .034, η² = 0.01. Students with formal AI training demonstrated significantly more positive attitudes, t(664) = 2.59, p = .010, Cohen’s d = 0.23.

### Practices related to artificial intelligence

The overall mean practice score was 3.32 ± 0.70, indicating moderate AI engagement Table 4.

**Table 4:** Practices related to artificial intelligence use.

Item	Practice Statement	Mean	SD
1	AI use for exam preparation	3.46	1.03
2	AI use for assignments	3.77	0.91
3	AI use for research	3.72	0.94
4	AI use for brainstorming	3.48	1.00
5	AI use for career guidance	2.90	1.16
6	AI use for grammar/spelling	2.92	1.14
7	AI use for skill development	3.00	1.12
**Overall Practice Score**	—	**3.32**	0.70

No statistically significant gender differences were observed, t(664) = 1.62, p =.106.

Practice scores did not differ across academic years, F(4, 661) = 0.87, p = .476. College affiliation was significantly associated with practice levels, F(2, 663) = 10.94, p < .001, η² = 0.03. Formal AI training was not significantly associated with practice frequency, t(664) = 0.24, p = .807.

Bonferroni-adjusted post hoc tests showed that second-year (MD = 0.56, *p* = .014) and third-year students (MD = 0.54, *p* = .028) had higher AI knowledge than first-year students. Medicine and Health Sciences students had higher knowledge (MD = 0.38, *p* = .027) and practice scores (MD = 0.20, *p* = .005) than Nursing students as in Table 5.

**Table 5:** Bonferroni-adjusted post hoc comparisons for significant ANOVA findings.

Outcome variable	Group comparison	Mean difference	Bonferroni-adjusted *p* value
Knowledge	Second year vs. First year	0.56	.014
Knowledge	Third year vs. First year	0.54	.028
Knowledge	Medicine and Health Sciences vs. Nursing	0.38	.027
Attitudes	Nursing vs. Dentistry	0.11	.033
Practices	Medicine and Health Sciences vs. Nursing	0.20	.005
Practices	Dentistry vs. Nursing	0.26	.001

## Discussion

This study assessed knowledge, attitudes, and practices (KAP) toward artificial intelligence (AI) among health sciences students in a Palestinian university. The main finding is that students demonstrated positive attitudes and moderate academic use of AI tools, despite limited foundational knowledge. This pattern suggests that AI is already being used in students’ academic work, but not necessarily supported by sufficient conceptual understanding of machine learning, deep learning, data requirements, and ethical implications.

The knowledge findings revealed an important educational gap. Although many students were aware of AI applications in healthcare and recognized barriers to AI implementation, fewer demonstrated understanding of basic AI concepts and machine/deep learning. This finding is consistent with previous international and regional studies showing that health sciences students often have general awareness of AI but limited technical and theoretical knowledge [12–15]. The significantly higher knowledge scores among students who had received formal AI training further support the importance of structured educational exposure. However, because this study used a cross-sectional design, this association should be interpreted cautiously and not as evidence of causal impact.

Students’ attitudes toward AI were generally positive, particularly regarding the importance of learning AI basics, its relevance to future professional practice, and the need to understand ethical implications. These findings align with global evidence indicating that students are increasingly receptive to AI integration in health professions education [5,14,24]. At the same time, some concern remained regarding professional replacement, diagnostic errors, and over-reliance on AI. Such concerns should not be interpreted only as resistance; rather, they may reflect critical awareness of the ethical and professional risks associated with healthcare technologies. Veras argued that fear and caution often accompany major technological transitions and may stimulate responsible innovation and ethical reflection [17].

The practice findings indicate that students commonly used AI tools for assignments, research, examination preparation, and brainstorming. This suggests that AI has become part of students’ academic learning routines. However, the moderate overall practice score indicates that AI use remains selective rather than fully integrated across all learning and professional-development activities [12,15,25]. Unlike knowledge and attitudes, formal AI training was not significantly associated with AI-related practice frequency. Therefore, the findings suggest that formal training may be more strongly related to what students know and how they perceive AI than to how frequently they use it in daily academic activities.

The present results are also consistent with emerging evidence on generative AI use in health sciences education. Veras et al. conducted a mixed-methods crossover randomized controlled trial and found that structured exposure to ChatGPT in health sciences education improved students’ usability perceptions and engagement with AI-supported learning [26]. Compared with that experimental evidence, the current cross-sectional study shows that students may already be engaging with AI tools in routine academic tasks, but without adequate structured preparation. This highlights the need to move from informal AI use toward guided, curriculum-based AI literacy.

The findings partly support the KAP framework, but they also show that the relationship between knowledge, attitudes, and practice may not be linear in rapidly evolving digital contexts. Students reported positive attitudes and moderate practice despite limited foundational knowledge [10,26]. This suggests that exposure to AI tools may precede formal knowledge development. While this may encourage innovation and openness, it also raises concerns about uncritical use, inaccurate outputs, algorithmic bias, academic integrity, and ethical decision-making [6,9,27]. Therefore, AI education should not focus only on technical skills but should also include critical appraisal, ethical governance, data privacy, transparency, and the limits of AI in clinical judgment.

In the Palestinian context, these findings are particularly relevant. Health professions education operates within resource-constrained conditions, and digital transformation remains uneven across institutions. Although AI may offer opportunities to strengthen learning, research, and future clinical decision-making, its use must be accompanied by structured guidance, institutional policy, and faculty development. The study findings therefore support the integration of AI literacy into health sciences curricula, with attention to local educational capacity, ethical standards, and the realities of Palestinian healthcare education.

### Strengths and limitations

This study benefits from a large sample size and representation across multiple health disciplines. However, several limitations must be acknowledged. The cross-sectional design precludes causal inference, and the use of convenience sampling may limit generalizability beyond the study institution. Self-reported practice measures may be subject to response bias. Additionally, while internal consistency reliability was acceptable, future research employing confirmatory factor analysis and longitudinal designs would strengthen psychometric and temporal validity.

### Implications for health professions education

Within the scope of these findings, integrating structured AI literacy into health sciences curricula may help bridge the observed gap between enthusiasm and foundational understanding. Educational strategies could consider:

1. Introducing foundational AI concepts relevant to healthcare applications;
2. Embedding ethical and governance discussions aligned with international guidance
3. Providing supervised experiential learning opportunities;
4. Supporting faculty development to ensure pedagogical readiness.

Such considerations should be framed as institutional opportunities rather than prescriptive national mandates, given the study’s single-site design.

## Conclusion

In conclusion, health sciences students demonstrated readiness and enthusiasm toward AI integration, yet lacked sufficient foundational knowledge to ensure safe, ethical, and effective use. Addressing this discrepancy through structured, contextually relevant AI education is essential for preparing a digitally competent healthcare workforce in Palestine. Aligning educational strategies with global standards while responding to local healthcare realities will be critical to harnessing the benefits of AI without compromising professional values or patient-centered care.

## Supporting information

S1 File STROBE statement. Completed STROBE checklist for reporting the cross-sectional study, indicating the relevant manuscript sections where each reporting item is addressed.
(DOCX)

S2 File Original data. Anonymized original dataset used for the statistical analyses in this study.
(XLSX)
