# Supplementary material for: Knowledge, attitudes, and practices toward artificial intelligence among health sciences students: A cross-sectional study in Palestine
Source: PLoS One. 2026 Jun 26;21(6):e0352785. doi: 10.1371/journal.pone.0352785 (PMC13308871; doi:10.1371/journal.pone.0352785)
Supplement: S1 File — Completed STROBE checklist for reporting the cross-sectional study, indicating the relevant manuscript sections where each reporting item is addressed. (DOCX) [file pone.0352785.s001.docx]

**STROBE Statement—Checklist of Items for Cross-Sectional Studies**

| **Section/Topic** | **Item No.** | **Recommendation** | **Reported on Page No.** |
| --- | --- | --- | --- |
| **Title and Abstract** | 1(a) | Indicate the study design with a commonly used term in the title or abstract | Title; Abstract (Methods) |
|  | 1(b) | Provide an informative and balanced summary of what was done and what was found | Abstract |
| **Introduction** | 2 | Explain the scientific background and rationale for the investigation | Introduction |
|  | 3 | State specific objectives, including prespecified hypotheses | End of Introduction |
| **Methods** | 4 | Present key elements of study design early in the paper | Methods – Study Design |
|  | 5 | Describe the setting, locations, and relevant dates | Methods – Study Setting |
|  | 6(a) | Give eligibility criteria and sources/methods of participant selection | Methods – Study Population |
|  | 6(b) | Describe matching criteria, if applicable | Not applicable |
|  | 7 | Clearly define all outcomes, exposures, predictors, confounders, and effect modifiers | Methods – Instrument Description |
|  | 8* | For each variable of interest, give sources of data and measurement methods | Methods – Data Collection Instrument |
|  | 9 | Describe efforts to address potential sources of bias | Methods – Sampling & Limitations in Discussion |
|  | 10 | Explain how study size was determined | Methods – Sample Size Considerations |
|  | 11 | Explain how quantitative variables were handled in analyses | Methods – Statistical Analysis |
|  | 12(a) | Describe all statistical methods, including those used to control for confounding | Methods – Statistical Analysis |
|  | 12(b) | Describe methods used to examine subgroups and interactions | Methods – Inferential Statistics |
|  | 12(c) | Explain how missing data were addressed | Methods – Data Preparation |
|  | 12(d) | If applicable, describe analytical methods taking account of sampling strategy | Convenience sampling described |
|  | 12(e) | Describe sensitivity analyses | Not applicable |
| **Results** | 13(a) | Report numbers of individuals at each stage of study | Results – Participant Characteristics |
|  | 13(b) | Give reasons for non-participation at each stage | Results – Recruitment Flow |
|  | 13(c) | Consider use of flow diagram | Described in text |
|  | 14(a) | Give characteristics of study participants | Table 1 |
|  | 14(b) | Indicate number of participants with missing data | Methods – Data Preparation |
|  | 15 | Report numbers of outcome events or summary measures | Tables 2–4 |
|  | 16(a) | Give unadjusted and adjusted estimates with precision | Results – Inferential Analysis |
|  | 16(b) | Report category boundaries when continuous variables categorized | Knowledge scoring described |
|  | 16(c) | Translate relative risk into absolute risk if relevant | Not applicable |
|  | 17 | Report other analyses (subgroups, interactions) | Results – Group Comparisons |
| **Discussion** | 18 | Summarize key results with reference to objectives | Discussion – First paragraph |
|  | 19 | Discuss limitations of study | Strengths and Limitations |
|  | 20 | Provide cautious interpretation of results | Discussion |
|  | 21 | Discuss generalizability | Discussion – Contextual Interpretation |
| **Other Information** | 22 | Give source of funding and role of funders | Funding Statement |

- أسفل النموذج
